# Comprehensive analysis reveals key genes and environmental toxin exposures underlying treatment response in ulcerative colitis based on *in-silico* analysis and Mendelian randomization

**DOI:** 10.18632/aging.205294

**Published:** 2023-12-04

**Authors:** Yizhou Huang, Jie Liu, Dingbao Liang

**Affiliations:** 1 Department of Gastroenterology, The PLA Navy Anqing Hospital, Anqing 246000, Anhui Province, China; 2 Department of Gastroenterology, Division of Life Sciences and Medicine, University of Science and Technology of China, Hefei 230026, Anhui Province, China

**Keywords:** ulcerative colitis, microarray, biomarker, genomics, bioinformatics

## Abstract

Background: UC is increasingly prevalent worldwide and represents a significant global disease burden. Although medical therapeutics are employed, they often fall short of being optimal, leaving patients struggling with treatment non-responsiveness and many related complications.

Materials and Methods: The study utilized gene microarray data and clinical information from GEO. Gene enrichment and differential expression analyses were conducted using Metascape and Limma, respectively. Lasso Regression Algorithm was constructed using glmnet and heat maps were generated using pheatmap. ROC curves were used to assess diagnostic parameter capability, while XSum was employed to screen for small-molecule drugs exacerbating UC. Molecular docking was carried out using Autodock Vina. The study also performed Mendelian randomization analysis based on TwoSampleMR and used CTD to investigate the relationship between exposure to environmental chemical toxicants and UC therapy responsiveness.

Results: Six genes (ELL2, DAPP1, SAMD9L, CD38, IGSF6, and LYN) were found to be significantly overexpressed in UC patient samples that did not respond to multiple therapies. Lasso analysis identified ELL2 and DAPP1 as key genes influencing UC treatment response. Both genes accurately predicted intestinal inflammation in UC and impacted the immunological infiltration status. Clofibrate showed therapeutic potential for UC by binding to ELL2 and DAPP1 proteins. The study also reviews environmental toxins and drug exposures that could impact UC progression.

Conclusions: We used microarray technology to identify DAPP1 and ELL2 as key genes that impact UC treatment response and inflammatory progression. Clofibrate was identified as a promising UC treatment. Our review also highlights the impact of environmental toxins on UC treatment response, providing valuable insights for personalized clinical management.

## INTRODUCTION

Ulcerative colitis (UC) is a chronic relapsing bowel disease that results in inflammation, mucosal injury, and fibrosis of the intestinal wall [1]. Bloody diarrhea, weight loss, and abdominal pain are the most common symptoms of UC [2]. Recently, UC has exhibited an increasing prevalence worldwide and carries a significant global burden of disease [3]. The administration of certain medications may indeed control the inflammatory process to some extent, alleviate related symptoms, and improve the patient’s quality of life [4]. However, such medical remedies prove to be far from optimal, as patients frequently experience a lack of response to treatment along with a gamut of correlated complications [4].

Aminosalicylates (5-ASA) are the cornerstone of the medical management of mild-to-moderate UC [5]. The drugs have localized anti-inflammatory activity in the colon and are available in various forms such as suppositories, enemas, and oral medications. Corticosteroids are effective in controlling moderate-to-severe UC but are not suitable for long-term use due to substantial side effects such as immunosuppression and osteoporosis [6]. Immunomodulators, including azathioprine, 6-mercaptopurine, methotrexate, and cyclosporin, are effective in controlling UC’s inflammation and reducing the need for corticosteroids [7]. However, the onset of the therapeutic effect of these drugs may take weeks to months. Biologic therapies like anti-TNF-alpha (infliximab, adalimumab, golimumab), anti-IL 12/23 (ustekinumab), and anti-α4β7 (vedolizumab) have optimized the management of UC in the last decade [8–12]. The mechanisms of action of these drugs target specific pro-inflammatory cytokines or cells, reducing inflammation, and improving disease outcomes. However, as the incidence of UC increases, there is a gradual rise in the number of patients with refractory UC, characterized by poor or no response to traditional drug therapy, prolonged disease duration, and recurrent episodes [13]. It is, therefore, crucial to delve into biomarkers that are linked to treatment response in individuals with UC, and to devise novel methods for improving response rates. Such efforts hold tremendous clinical relevance and possess the potential to enhance patient outcomes, reduce symptom severity, and improve overall quality of life.

While previous research has identified several factors that contribute to the development and progression of UC, including dietary habits, lifestyle factors, and exposure to environmental toxins [14–17]. However, the role of genetic factors and environmental toxin exposures influencing therapeutic response in UC remains largely unexplored. In recent years, the proliferation of high-throughput sequencing and bioinformatics analysis has enabled the expeditious and precise detection of heterogeneity in scores of diseases [18–22]. Consequently, bioinformatics analysis has potential to be instrumental in identifying diverse gene expression patterns among UC patients with varying treatment response levels, thereby paving the way for the development of novel biomarkers as well as innovative treatment modalities.

In this context, our study aims to conduct a comprehensive analysis of the genetic factors contributing to the therapeutic responsiveness of UC patients. Our investigation endeavors to incorporate a comprehensive array of microarray data concerning the response of UC treatment after the administration of diverse pharmacotherapies to generate a more all-inclusive Pan-therapy Analysis. Our findings have the potential to inform clinical decision-making in UC treatment and identifying patients who may benefit most from targeted intervention strategies. Ultimately, we hope that our study contributes to the broader efforts of developing more effective and personalized treatments for UC.

## MATERIALS AND METHODS

### Data acquisition

In this study, we acquired high-throughput sequencing data for ulcerative colitis, along with its clinical information, from the Gene Expression Omnibus (GEO) database [23]. To ensure that the dataset we used met our research criteria, we implemented a rigorous selection process based on four criteria. Firstly, we limited our selection to specimens that were intestinal mucosal tissues. Secondly, we ensured that the clinical information included details of therapeutic drugs that had already been approved by the FDA for clinical use in ulcerative colitis patients. Thirdly, we only included clinical data for patients who had demonstrated a response to the administered medication. Fourthly, we required follow-up information on the effectiveness of the drugs to be available for at least one month.

Following this selection process, we obtained five datasets that met our inclusion criteria and provided information regarding the therapeutic efficacy of four distinct medications: GSE109142, GSE92415, GSE12251, and GSE14580. Specifically, we found that in the GSE109142 cohort, 53 individuals were treated with 5-aminosalicylic acid (5ASA), while 153 individuals received corticosteroid treatment. Additionally, in the GSE92415 cohort, 109 individuals were treated with golimumab. For the GSE12251 and GSE14580 cohorts, treatment response information for 23 and 24 patients, respectively, all treated with infliximab, was available.

To merge the GSE12251 and GSE14580 datasets, we employed the R software package inSilicoMerging, and then applied the method proposed by Johnson WE et al. to eliminate batch effects [24–26]. This resulted in a matrix (GSE12251 and GSE14580) that was free of such effects, and suitable for further analysis.

### Differential gene expression analysis and least absolute shrinkage and selection operator (Lasso) regression algorithm

In this study, we used the R package Limma to identify differential expression genes (DEGs) between drug-responsive and non-responsive samples [27, 28]. A fold change (FC) greater than 1.2 or less than 0.8 and a p-value less than 0.05 were used to select DEGs [29–32]. We conducted Limma analysis on GSE109142, GSE92415, and the merged dataset GSE12251 and GSE14580 separately and obtained candidate hub genes by taking the intersection of all the differentially expressed genes (DEGs). Next, we incorporated these candidate hub genes into the analysis of the Lasso Regression Algorithm, which was executed using the R package glmnet [33–36]. The Lasso Regression Algorithm eliminates collinear genes, with default parameter values [37, 38]. We identified a set of key genes associated with the responsiveness of all drugs included in this study by intersecting the genes obtained from different datasets using the Lasso Regression Algorithm. A heat map was created using the R package “pheatmap” [39].

### Evaluation of immune cell infiltration status

The immune cell infiltration status of all samples was evaluated using the online network tool Immune Cell Abundance Identifier (ImmuCellAI), which provides a comprehensive analysis of 18 T-cell subtypes and 6 other immune cell types, including B cells, NK cells, monocytes, macrophages, neutrophils, and DC cells. The input file for the analysis consisted of the complete gene expression profiles of each dataset [40, 41]. The specific URL for ImmuCellAI is http://bioinfo.life.hust.edu.cn/ImmuCellAI#!/.

### Prediction of the biological function

GeneMANIA (http://www.genemania.org) is a web-based platform that facilitates the construction of protein-protein interaction (PPI) networks, offering hypotheses on gene function prediction and identification of genes that have similar roles [42]. The network integration algorithm incorporates various bioinformatics techniques such as physical interaction, co-expression, colocalization, gene enrichment analysis, genetic interaction, and website prediction. In this study, the PPI was analyzed using GeneMANIA to predict the biological function of individual genes. GeneMANIA performs Gene Ontology (GO) enrichment analysis on genes in the interaction network, and corrects the p-values using the Benjamini-Hochberg procedure [43]. Categories are presented up to a false discovery rate (FDR) cutoff of 0.01.

Enrichment analysis of biological functions for a set of genes was carried out using the Metascape database [44]. All parameters except those specified were set to default values. In the next step of the analysis, terms with a p-value less than 0.01, a minimum count of 3, and an enrichment factor greater than 1.5 were selected. Applying screening criteria of kappa scores equal to 4 and similarity greater than 0.3, enrichment terms were categorized into clusters by Metascape using hierarchical clustering. Term representatives were chosen based on minimum p-values.

### Chemical-gene interaction analysis

Based on the meticulously curated research on Comparative Toxicogenomic Database (CTD), we analyzed the relationship between environmental chemical toxicant exposure and the responsiveness to UC therapy [45]. Our analysis examined all previously identified key genes for their effects on gene expression caused by environmental toxicants and drugs. The analysis was not restricted to any particular species [46].

### Discovery of potential drugs by computational methods

The eXtreme Sum (XSum) algorithm was employed to identify potential small molecule drugs from the Connectivity Map (CMAP) database using a similarity scoring approach [47]. The input files consisted of the top 150 upregulated and top 150 downregulated genes, and the XSum algorithm calculated a score for each patient, with a lower score indicating a higher potential for a drug to act as a therapeutic agent. Using the Protein Data Bank (PDB) at the RCSB (https://www.rcsb.org/pdb/home/home.do), the crystal structures of hub gene proteins were determined. PubChem was used to download the 3D structures of the small molecule drugs (https://www.ncbi.nlm.nih.gov/pccompound) [48, 49]. Autodock Vina was used for molecular docking, which involved the preparation of proteins and ligands, setting up a grid, and docking the compounds [50]. We selected the best pose based on the docking score and the rationality of the molecular conformation.

### Data sources of mendelian randomization (MR) analysis

Our research utilizes summary-level data derived from IEU Open GWAS database (https://gwas.mrcieu.ac.uk) with a MR approach. It’s noteworthy that the IEU Open GWAS database consists of study participants who have provided informed consent in their respective studies. The GWAS data for DAPP1 and ELL2 were obtained from GWAS ID: eqtl-a-ENSG00000070190 and GWAS ID: eqtl-a-ENSG00000118985, respectively. The UC GWAS database (GWAS ID: ieu-a-1126) provided us with information on 463,010 individuals of European ancestry, including 1,987 cases and 461,023 controls.

### Instrumental variable (IVs) selection

Instrumental variables (IVs), using genetic variations, are leveraged in MR to obtain unbiased estimations of the causal impact of the exposure variable of interest on an outcome variable. Firstly, we identified single-nucleotide polymorphisms (SNPs) significantly associated with the exposure variable (p < 5*10-8) to serve as IVs for MR analysis. The clumping technique was employed to address any linkage disequilibrium issues between the selected SNPs. Furthermore, sensitivity analysis was undertaken to validate the robustness of the identified IVs. A clumping method with a window size of 10,000kb and R2 value below 0.001 was utilized to eliminate SNPs indicating significant Linkage Disequilibrium (LD). To ensure the reliability of our data, we utilized the Phenoscanner database (http://www.phenoscanner.medschl.cam.ac.uk/) to investigate the likely correlation of included SNPs with confounding variables and outcomes of interest (p < 5X10-8). MR-PRESSO was also applied to our results for excluding outliers and incorporating horizontal pleiotropy. Additionally, the F-statistics were calculated cumulatively for SNPs using the following formula: F = (N-k-1)R2/k(1-R2), where R2 represents the variation in the exposure explained by each IV. The strength of the instrument was evaluated using the F-statistics, where an F value above 10 is indicative of adequate statistical power.

### MR analysis

In the quest to verify the causal connection between exposure and outcome, our study conducted MR analysis using R Version 4.2.1 and the “TwoSampleMR” package, utilizing multiple MR methods, including the inverse variance weighted (IVW), the weighted median (WM), the MR-Egger method, simple mode, and weighted model. The IVW approach was predominantly utilized as it provides superior statistical validity compared to other available methods and can estimate the causal impact of exposure on the outcome consistently.

### Real time quantitative PCR (RT-qPCR)

The present study describes the methodology employed for total RNA extraction, cDNA synthesis, and gene expression quantification using RT-qPCR assay. RNA was isolated using TRIzol reagent (Ambion, USA), while cDNA was synthesized using PrimeScriptTM RT Master Mix (Takara, Japan). We measured the relative expression levels of DAPP1, ELL2, and GAPDH genes using ChamQ SYBR qPCR Master Mix (Vazyme, China) and the 2-ΔΔCT method. The specificity of the RT-qPCR reaction was ensured by using gene-specific primers designed for DAPP1, ELL2, and GAPDH genes. The results were evaluated based on GAPDH as the internal reference gene, and the experiment was performed in triplicates for establishing the average value. Our findings demonstrate the successful detection of gene expression levels through RT-qPCR assay. To detect DAPP1, ELL2 and GAPDH expression levels, the forward primer of DAPP1 was 5′-GGTTACCTCACCAAACAGGGA-3′, and the reverse primer of DAPP1 was 5′-GGTTCTGGTGACATCTGGTCTT-3′; the forward primer of ELL2 was 5′-TGACTGCATCCAGCAAACAT-3′, and the reverse primer of ELL2 was 5′-TCGTTTGTTGCACACACTGTAA-3′; while the forward primer of GAPDH was 5′-CATGTTCGTCATGGGTGTGA-3′ and the reverse primer of GAPDH was 5′-GGTGCTAAGCAGTTGGTGGT-3′. The current research utilized samples obtained from eight UC patients, who were admitted to The PLA Navy Anqing Hospital. The samples were subjected to RT-qPCR analysis as per established protocols. Informed consent was obtained from all patients involved in the study.

### Statistics

The statistical analyses were conducted using R software (version 4.2.1). Continuous variables were compared using the Wilcoxon test, while differences in proportions were evaluated using the chi-square test. Statistical significance was defined as a p-value below 0.05. A Receiver Operating Characteristic (ROC) curve was generated to evaluate the predictive performance. The correlation analysis was conducted using Spearman’s correlation coefficient.

### Data availability statement

Publicly available datasets were analyzed in this study. This data can be found in Gene Expression Omnibus (GEO) database (https://www.ncbi.nlm.nih.gov/geo/).

## RESULTS

### Key genes influencing therapeutic response in UC uncovered through Limma and Lasso analyses

**Figure 1:**
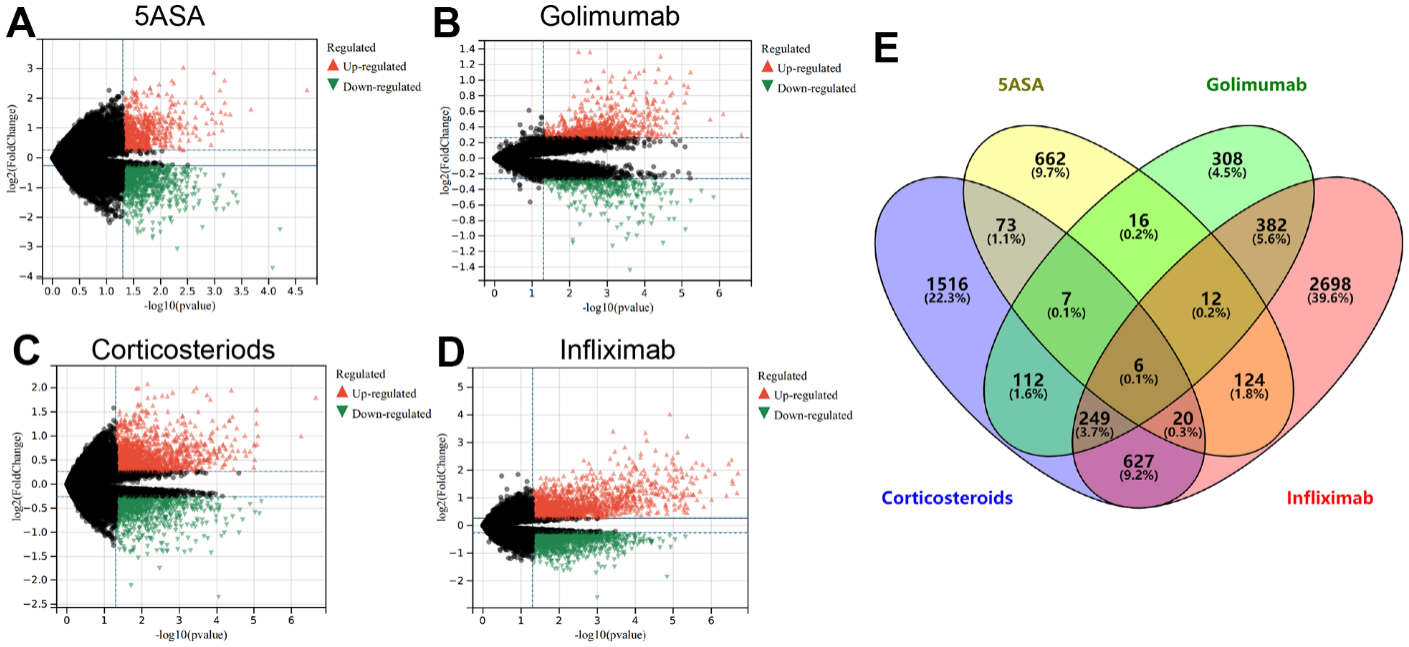
The volcano plot depicts the upregulated (in red) and downregulated (in green) genes observed in UC patients who remain unresponsive to treatment with 5-ASA (**A**), golimumab (**B**), corticosteroids (**C**), or infliximab (**D**). (**E**) The Venn diagram exhibits genes that are differentially expressed across all treatment modalities.

Using Limma analysis, we identified DEGs between non-responding and responding UC patients treated with 5-ASA, corticosteroid, golimumab, and infliximab (Supplementary Table 1). In individuals unresponsive to 5-ASA treatment, 163 genes were upregulated, while 757 genes were downregulated, as shown in Figure 1A. For golimumab, 699 genes were upregulated, while 402 genes were downregulated (Figure 1B). Notably, 1,057 upregulated genes and 1,553 downregulated genes were observed in corticosteroid non-responders (Figure 1C). Similarly, infliximab unresponsive patients exhibited upregulation of 1,758 genes and downregulation of 2,400 genes (Figure 1D). Supplementary Figure 1 presents a heatmap representation of the top 20 differentially upregulated and downregulated genes. Using a Venn diagram to perform an intersection analysis of the DEGs, six hub genes, namely ELL2, DAPP1, SAMD9L, CD38, IGSF6, and LYN, were consistently found to be upregulated in all treatment-resistant patients.

**Figure 2:**
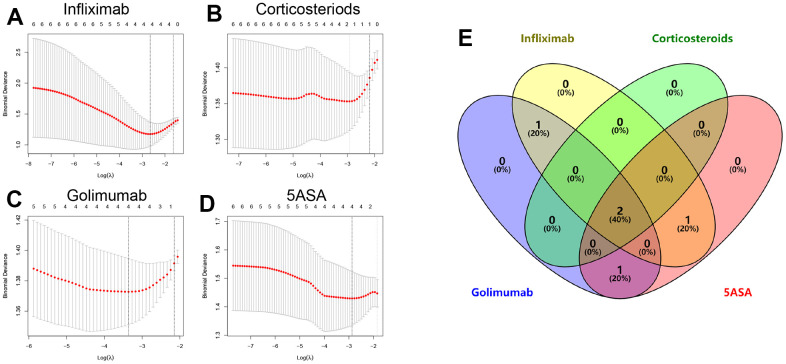
Lasso regression analysis results and partial likelihood deviance for the Lasso regression in infliximab (**A**), corticosteroids (**B**), golimumab (**C**), or 5-ASA (**D**) treatment cohorts. (**E**) Venn diagram showing overlapping key genes in Lasso regression.

Using the expression profiles of the six hub genes mentioned above from patient samples subjected to distinct treatments as input files, we conducted a Lasso analysis to remove collinear genes (Figure 2A–2D and Supplementary Table 2). Intersecting the Lasso analysis outcomes, we identified a pair of genes (DAPP1 and ELL2) that were the key genes in modulating drug response of UC patients to 5-ASA, corticosteroid, golimumab, and infliximab (Figure 2E). We categorized patients according to the median levels of DAPP1 (ELL2) expression. Regardless of the therapeutic intervention used, patients grouped as high DAPP1 (ELL2) exhibited a significantly higher frequency of treatment non-responsiveness (Supplementary Figure 2).

### Immune infiltration between different treatment responses

**Figure 3:**
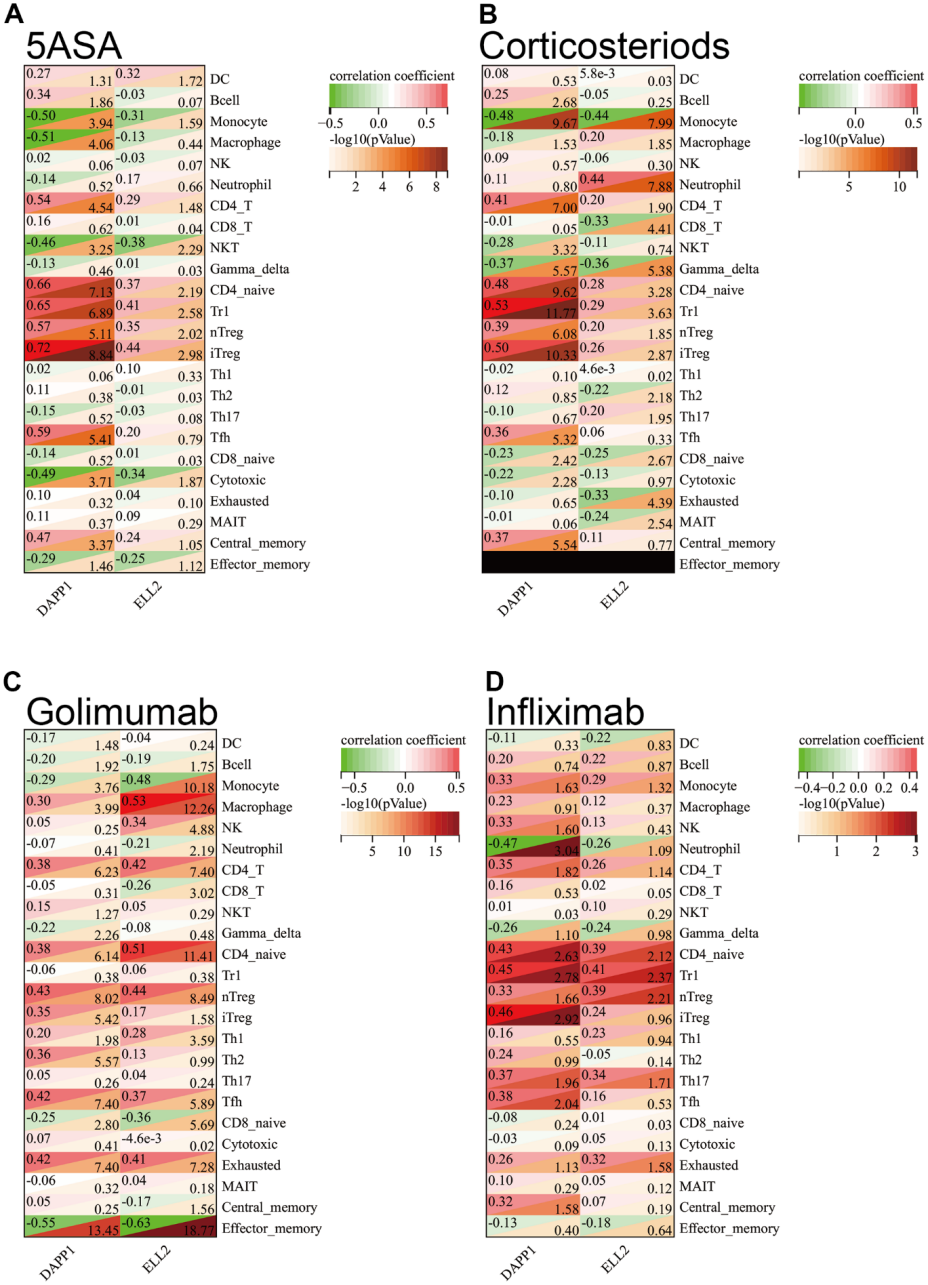
The Spearman correlation analysis demonstrated the correlation between gene expression levels of DAPP1 and ELL2 and the extent of immune cell infiltration in cohorts of UC patients who received 5-ASA (**A**), corticosteroids (**B**), golimumab (**C**), or infliximab (**D**), respectively.

In order to gain deeper insights into the factors affecting the responsiveness of UC drug therapy due to DAPP1 and ELL2, our investigation centered around the dissimilarities in the infiltration status of immune cells. We evaluated the correlation between DAPP1 and ELL2 expression and the extent of immune cell infiltration in each cohort of UC patients after every treatment. The correlation between DAPP1 and ELL2 with immune cell infiltration was notable, particularly for DAPP1, which exhibits a strong positive correlation (correlation coefficient R =0.72, P<0.001) with iTreg cells in samples receiving 5ASA treatment (Figure 3A–3D). Supplementary Figure 3 depicted the alterations in the proportions of various immune cell infiltrates in response to the upregulation of DAPP1 or ELL2 gene expression levels within the sample.

**Figure 4:**
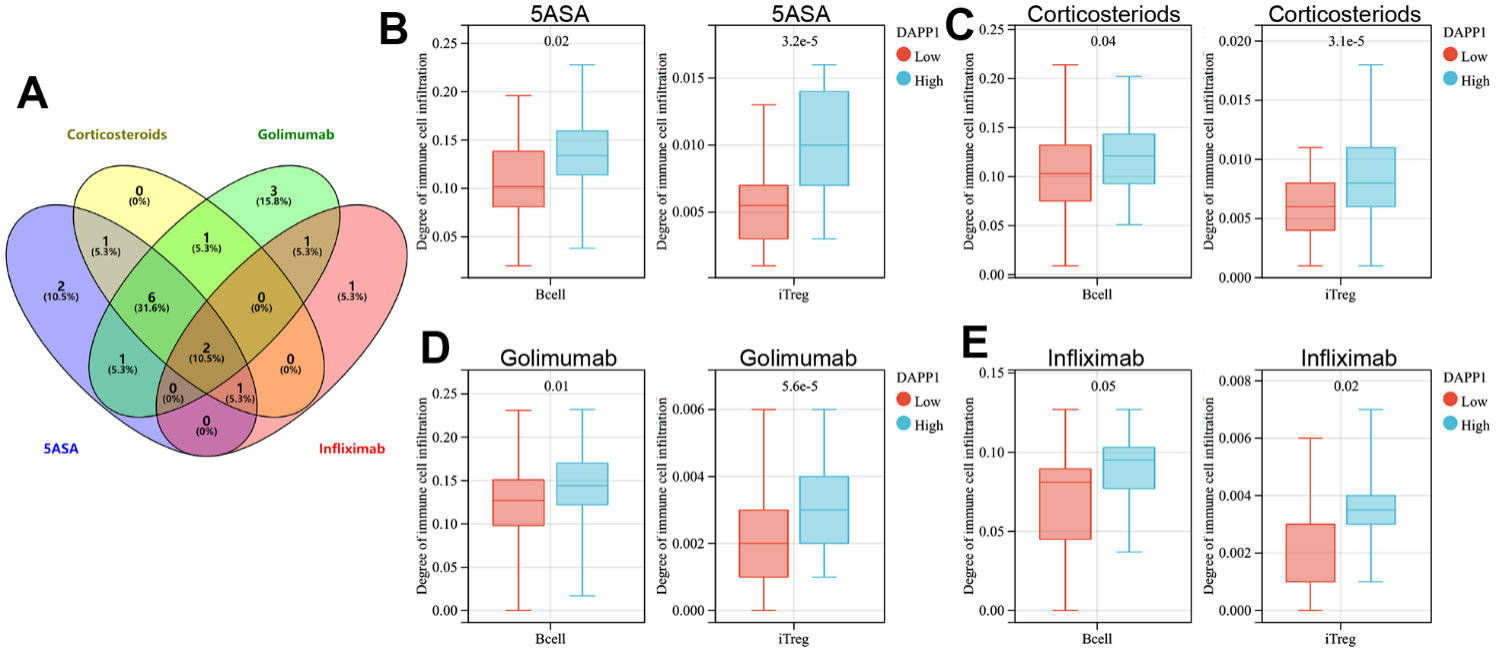
(**A**) The Venn diagram displays immune cells with differential infiltration between high and low levels of DAPP1 expression across all treated cohorts. The differences in infiltration of B cells and iTregs between patients with high and low levels of DAPP1 in UC cohorts treated with 5-ASA (**B**), corticosteroid (**C**), golimumab (**D**), and infliximab (**E**).

**Figure 5:**
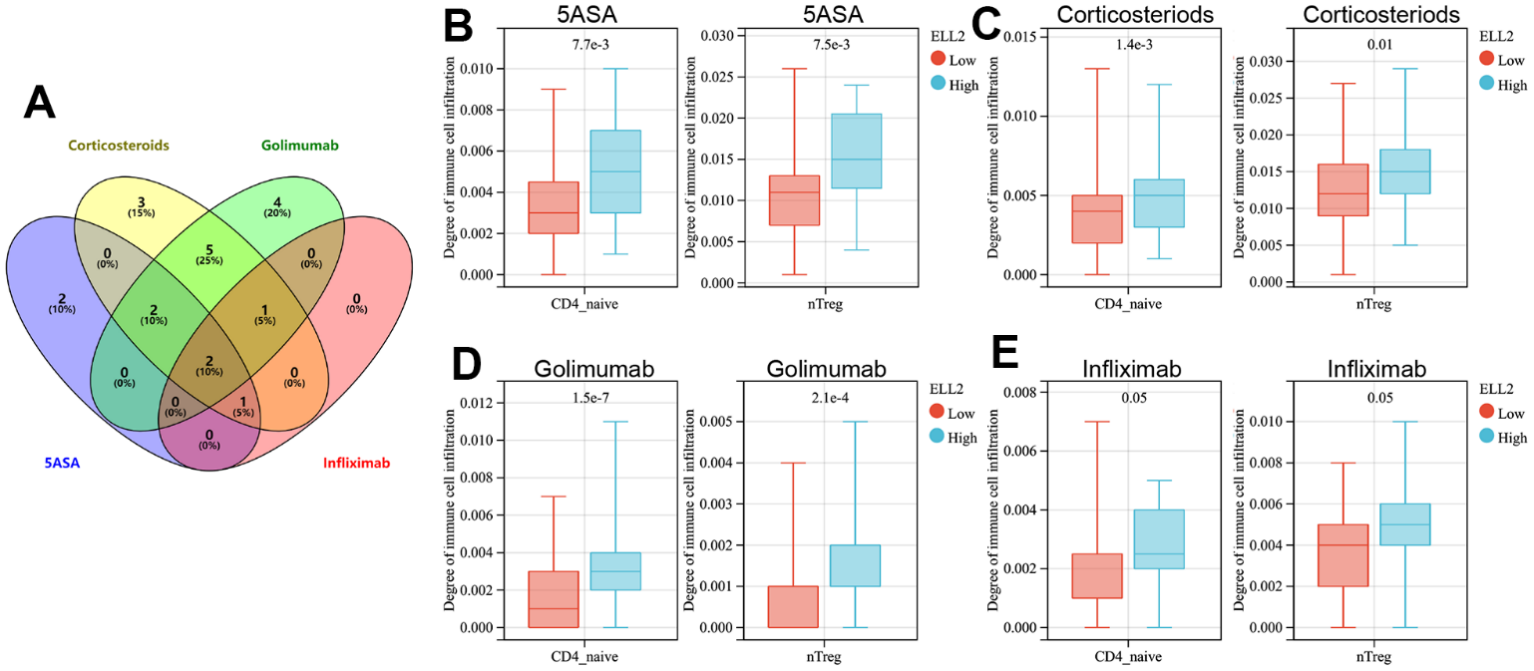
(**A**) The Venn diagram displays immune cells with differential infiltration between high and low levels of ELL2 expression across all treated cohorts. The differences in infiltration of B cells and iTregs between patients with high and low levels of ELL2 in UC cohorts treated with 5-ASA (**B**), corticosteroid (**C**), golimumab (**D**), and infliximab (**E**).

We then explored the immune cell types affected by both DAPP1 and ELL2 across all treatment modalities. The immunocyte types demonstrating significant differences in infiltration levels between UC patients with high and low DAPP1 (ELL2) after treatments with 5-ASA, corticosteroid, golimumab, or infliximab were presented in Supplementary Table 3. The Venn diagram suggested that regardless of the treatment, the expression level of DAPP1 affects the infiltration levels of B cells and iTreg cells (Figure 4A). Patients with elevated DAPP1 gene expression levels exhibit significantly higher levels of infiltration of B cells and iTreg cells (Figure 4B–4E). The Venn diagram also illustrated that the expression level of ELL2 impacts the infiltration levels of CD4-naive and nTreg cells, regardless of the administered treatment (Figure 5A). Notably, patients displaying heightened expression levels of ELL2 demonstrate a marked increase in infiltration levels of both CD4-naive and nTreg cells, as shown in Figure 5B–5E.

### Prediction of the biological function of DAPP1 and ELL2

**Figure 6:**
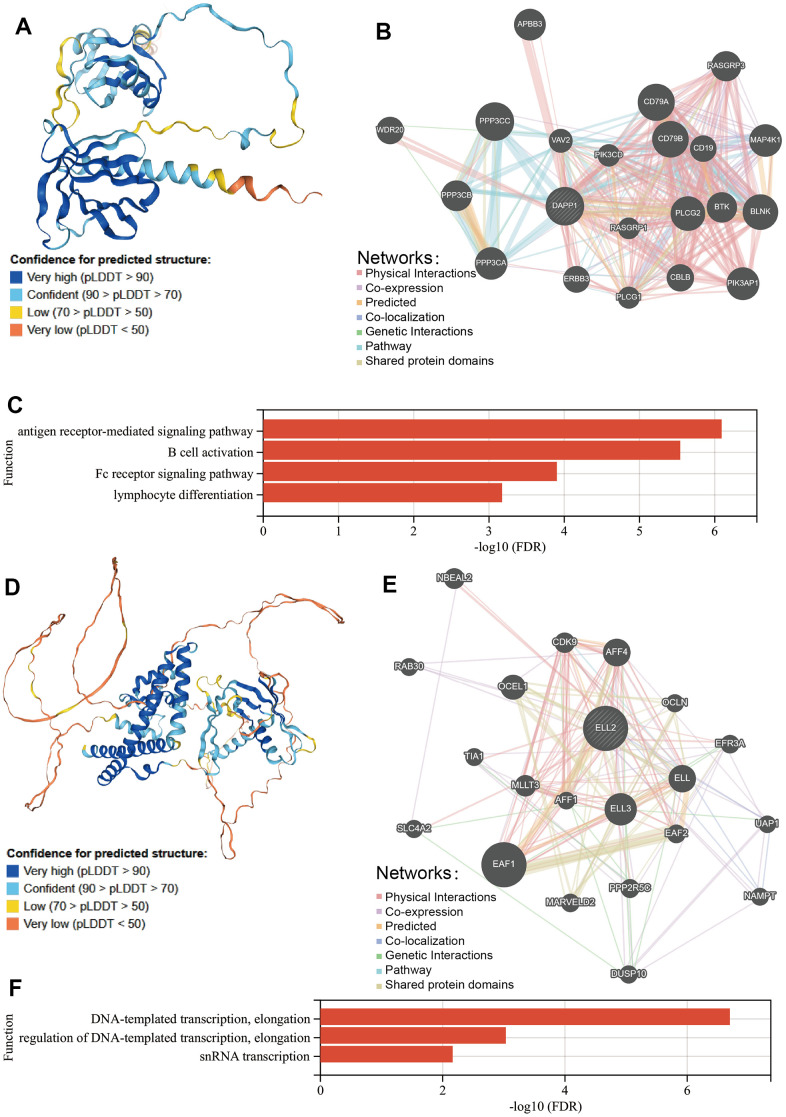
(**A**) The structure of the DAPP1 protein from HPA database. (**B**) The proteins that interact with DAPP1 obtained from GeneMANIA database. (**C**) Prediction of the biological function of DAPP1 based on GeneMANIA database. (**D**) The structure of the ELL2 protein from HPA database. (**E**) The proteins that interact with ELL2 obtained from GeneMANIA database. (**F**) Prediction of the biological function of ELL2 based on GeneMANIA database.

We utilized the Human Protein Atlas (HPA) database to illustrate the structure of the DAPP1 protein, as shown in Figure 6A. Subsequently, we employed the GeneMANIA database to explore the proteins that interact with DAPP1, resulting in a total of twenty proteins (Figure 6B). All types of interactions among these proteins have been summarized in Supplementary Table 4. GeneMANIA also performed a gene enrichment analysis on these twenty interacting proteins and DAPP1, revealing significant enrichment in four biological processes including antigen receptor-mediated signaling pathway, B cell activation, Fc receptor signaling pathway, and lymphocyte differentiation (Figure 6C). These results support our previous findings that DAPP1 impacts the infiltration levels of B cells and iTreg cells.

Then we utilized the HPA database to depict the structure of the ELL2 protein, which is depicted in Figure 6D. Employing the GeneMANIA database, we then investigated the interacting proteins of ELL2, which were found to total twenty (Figure 6E and Supplementary Table 4). Through gene enrichment analysis via GeneMANIA, we found that these twenty interacting proteins and DAPP1 were significantly enriched in three distinct biological processes: DNA-templated transcription, elongation, regulation of DNA-templated transcription, elongation, and snRNA transcription.

### Predictive capacity of DAPP1 and ELL2 for inflammatory status in UC colonic

Accurately predicting and monitoring the inflammatory status of UC colonic is crucial for managing the condition. In this study, we aimed to investigate the predictive ability of DAPP1 and ELL2 regarding the inflammatory status of UC colonic. By analyzing the predictive capacity of these biomarkers, we can gain more insight into their potential utility in clinical interventions for managing UC. The GSE179285 sequencing dataset consists of intestinal mucosal specimens obtained from patients with Inflammatory Bowel Disease (IBD), including 31 control samples, 55 samples with UC, and 168 samples with Crohn’s disease (CD). The inflammatory status of each IBD patient’s intestines was assessed and classified into two categories: Uninflamed and Inflamed.

**Figure 7:**
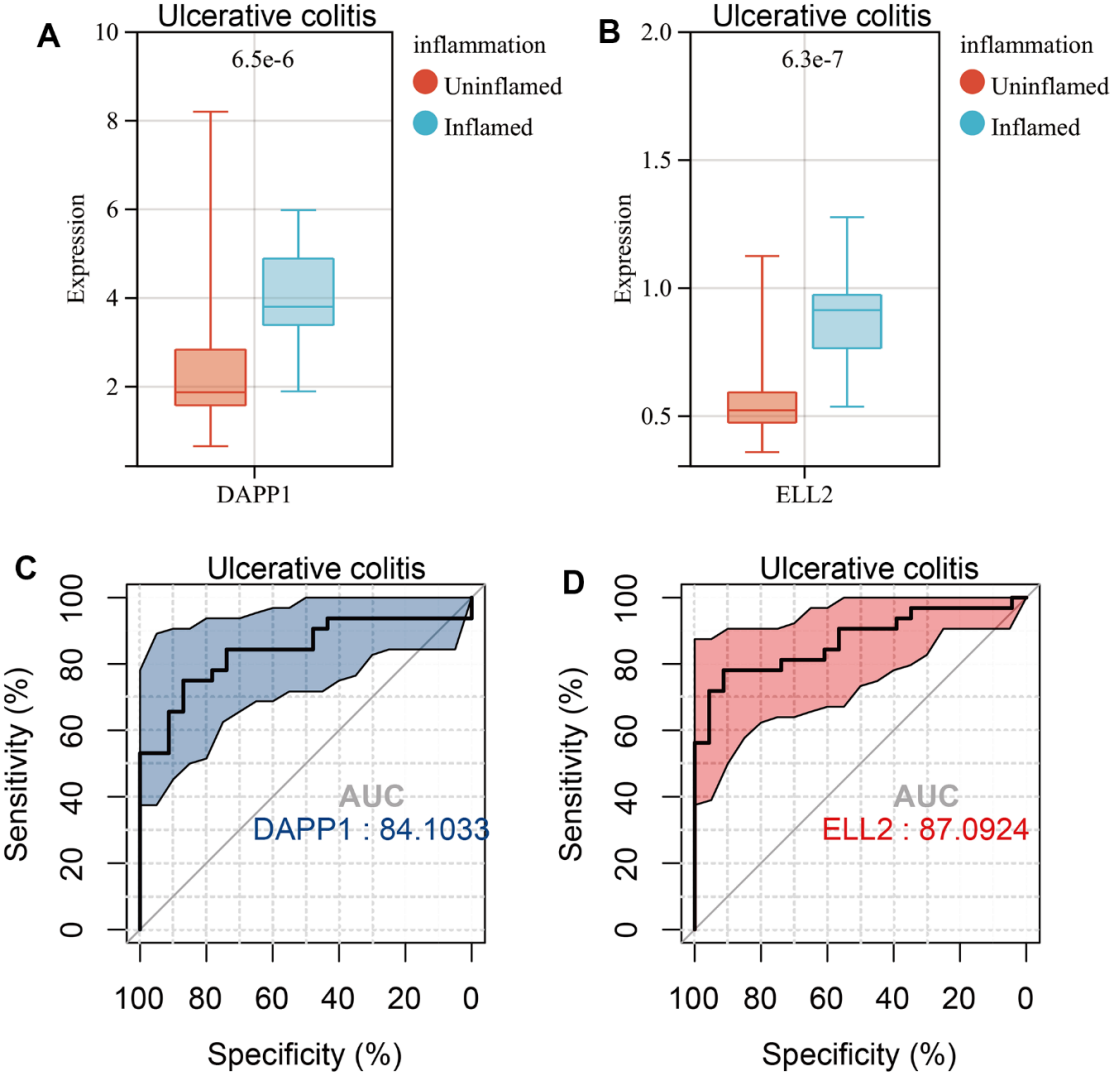
Boxplots of the gene expression levels of DAPP1 (**A**) and ELL2 (**B**) in inflamed and uninflamed UC samples in GSE179285 cohort. AUCs of DAPP1 (**C**) and ELL2 (**D**) in ROC analysis predicting Inflamed UC samples.

The Wilcoxon test indicated significant upregulation of DAPP1 and ELL2 in Inflamed UC samples (Figure 7A, 7B). DAPP1 and ELL2 showed excellent predictability for intestinal inflammation in UC, with respective AUC scores of 0.841 and 0.871 (Figure 7C, 7D). Similarly, DAPP1 and ELL2 were upregulated in Inflamed Crohn’s disease samples (Supplementary Figure 4A, 4B). ROC analysis demonstrated excellent predictive ability of DAPP1 and ELL2 for intestinal inflammation in Crohn’s disease, with AUCs exceeding 0.8 (Supplementary Figure 4C, 4D). Additionally, our study suggested that DAPP1 and ELL2 may serve as promising biological markers for IBD diagnosis, since we observed significant elevation in their expression levels in IBD samples compared to those from healthy controls (Supplementary Figure 4E, 4F).

### Exploring potential small molecule drugs for the alleviation of UC inflammation

In GSE179285, we employed the Limma analysis to explore DEGs between Uninflamed and Inflamed UC (Supplementary Table 5). Using the top 150 upregulated and top 150 downregulated genes in Inflamed UC as input for XSum algorithm, we calculated XSum scores for all small molecule drugs in the CMap database (Supplementary Table 6). The 300 genes used as input files were subjected to biological process enrichment analysis in the Metascape database. The enrichment of these 300 genes is primarily noticeable in the biological processes of immune response and inflammatory reaction, comprising NABA MATRISOME ASSOCIATED, humoral immune response, inflammatory response, Bile secretion, and Interleukin-4 and Interleukin-13 signaling (Supplementary Figure 5).

**Figure 8:**
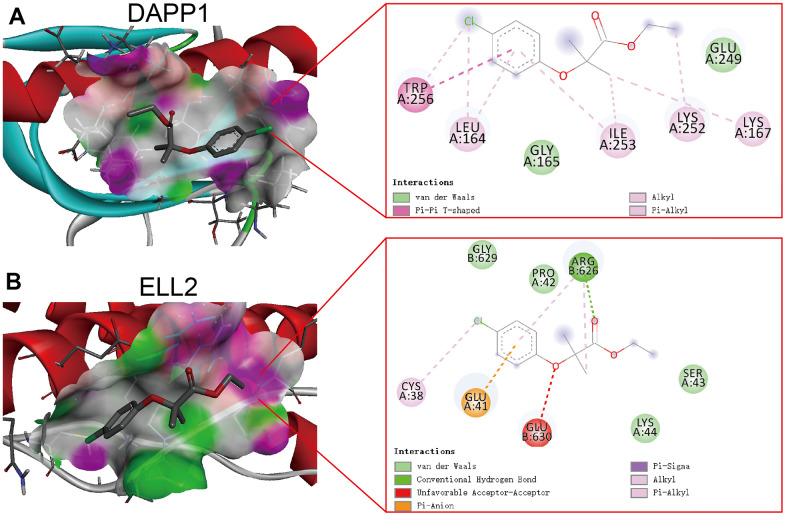
The optimal poses of the molecular docking between Clofibrate and DAPP1 (**A**) and ELL2 (**B**) presenting in both three-dimensional and two-dimensional formats.

Clofibrate obtained the lowest XSum score, suggesting it holds the greatest potential for reversing the Inflamed state of UC colonic. The chemical formula and structure of Clofibrate are presented in Table 1. To explore the potential targets for the therapeutic efficacy of Clofibrate, we performed molecular docking of Clofibrate and DAPP1 and ELL2. The optimal poses of the molecular docking were displayed in three-dimensional and two-dimensional formats in Figure 8A, 8B, respectively. The affinity scores of the molecular docking between Clofibrate and both DAPP1 and ELL2 were -4.9 kcal/mol and -5.2 kcal/mol, respectively, indicating a relatively favorable binding. Therefore, Clofibrate also has the potential to become an adjuvant therapeutic drug for UC patients who are unresponsive to 5-ASA, corticosteroids, golimumab, and infliximab, although further experimental research is necessary.

**Table 1:** Chemical structure formulae of clofibrate.

**Tag**	**Description**
**PubChem CID**	2796
**Structure**	
**Molecular Formula**	C_12_H_15_ClO_3_
**InChI**	1S/C12H15ClO3/c1-4-15-11(14)12(2,3)16-10-7-5-9(13)6-8-10/h5-8H,4H2,1-3H3 clofibrate 637-07-0
**Synonyms**	Ethyl 2-(4-chlorophenoxy)-2-methylpropanoate Ethyl clofibrate Atromid
**Molecular Weight**	242.70

### Exploration of environmental toxin exposures that impact therapeutic responsiveness in UC

The CTD database was used to investigate all potential Environmental Toxin Exposures that might affect DAPP1 and ELL2 gene expression levels, including methylation (Supplementary Table 7). In total, 17 types of environmental toxins were identified that could influence the methylation state and expression levels of DAPP1 and ELL2 genes, including 1,2-Dimethylhydrazine, Asbestos (Crocidolite), Benzo(a)pyrene, Bisphenol A, Dibutyl Phthalate, Fipronil, Hydrogen Peroxide, Carbon Nanotubes, Nickel, Particulate Matter, Silicon Dioxide, Sodium Arsenite, Sulforaphane, T-2 Toxin, Tetrachlorodibenzodioxin, Tobacco Smoke Pollution, and Vehicle Emissions (Table 2). Therefore, these Environmental Toxin Exposures could potentially influence the therapeutic response of UC patients, a response that is mediated by the intermediary factors DAPP1 and ELL2.

**Table 2:** The interaction between environmental toxin exposure and DAPP1 and ELL2.

**Chemical name**	**Gene symbol**	**Interaction actions**	**Organism**	**Reference pubMedID**
1,2-Dimethylhydrazine	DAPP1	affects response to substance|increases expression	Rattus norvegicus	27840820
Asbestos, Crocidolite	DAPP1	decreases expression	Homo sapiens	29523930
Benzo(a)pyrene	DAPP1	decreases methylation	Homo sapiens	27901495
Benzo(a)pyrene	DAPP1	decreases methylation	Homo sapiens	27901495
Benzo(a)pyrene	DAPP1	increases methylation	Homo sapiens	27901495
Benzo(a)pyrene	DAPP1	affects expression|affects reaction	Mus musculus	22228805
Benzo(a)pyrene	DAPP1	decreases expression	Mus musculus	19770486
Benzo(a)pyrene	DAPP1	increases expression	Mus musculus	22228805
Bisphenol A	DAPP1	affects cotreatment|increases methylation	Homo sapiens	31601247
Bisphenol A	DAPP1	affects expression	Rattus norvegicus	25181051
Dibutyl Phthalate	DAPP1	decreases expression	Mus musculus	21266533
Fipronil	DAPP1	increases expression	Rattus norvegicus	23962444
Hydrogen Peroxide	DAPP1	affects expression	Homo sapiens	21179406
Nanotubes, Carbon	DAPP1	increases expression	Mus musculus	25554681
Nanotubes, Carbon	DAPP1	increases expression	Mus musculus	25554681
Nickel	DAPP1	increases expression	Homo sapiens	25583101
Particulate Matter	DAPP1	increases expression	Homo sapiens	29703138
Silicon Dioxide	DAPP1	increases expression	Homo sapiens	25351596
Silicon Dioxide	DAPP1	decreases expression	Mus musculus	19073995
sodium arsenite	DAPP1	affects expression	Homo sapiens	29319823
Sulforaphane	DAPP1	decreases expression	Homo sapiens	31838189
T-2 Toxin	DAPP1	increases expression	Gallus gallus	31299295
Tetrachlorodibenzodioxin	DAPP1	affects expression	Mus musculus	21570461
Tetrachlorodibenzodioxin	DAPP1	decreases expression	Mus musculus	19770486
Tetrachlorodibenzodioxin	DAPP1	increases expression	Mus musculus	19465110
Tetrachlorodibenzodioxin	DAPP1	decreases expression	Rattus norvegicus	34747641
Tetrachlorodibenzodioxin	DAPP1	increases expression	Rattus norvegicus	32109520|33387578
Tobacco Smoke Pollution	DAPP1	increases expression	Homo sapiens	33660061
Tobacco Smoke Pollution	DAPP1	affects expression	Mus musculus	20133372
Vehicle Emissions	DAPP1	affects response to substance	Mus musculus	31869344
1,2-Dimethylhydrazine	ELL2	decreases expression	Mus musculus	22206623
Asbestos, Crocidolite	ELL2	increases expression	Homo sapiens	18687144
Asbestos, Crocidolite	ELL2	decreases expression	Mus musculus	29279043
Benzo(a)pyrene	ELL2	decreases expression	Homo sapiens	26238291
Benzo(a)pyrene	ELL2	decreases methylation	Homo sapiens	27901495
Benzo(a)pyrene	ELL2	increases methylation	Homo sapiens	27901495
Bisphenol A	ELL2	affects expression	Danio rerio	21786754
Bisphenol A	ELL2	affects expression	Homo sapiens	20170705
Bisphenol A	ELL2	decreases expression	Homo sapiens	22576693
Bisphenol A	ELL2	increases expression	Homo sapiens	25047013
Bisphenol A	ELL2	increases methylation	Homo sapiens	22576693
Bisphenol A	ELL2	increases expression	Mus musculus	25594700
Bisphenol A	ELL2	affects expression	Pimephales promelas	21786754
Bisphenol A	ELL2	decreases expression	Rattus norvegicus	25181051
Dibutyl Phthalate	ELL2	increases expression	Mus musculus	21266533
Fipronil	ELL2	affects expression	Danio rerio	32977147
Hydrogen Peroxide	ELL2	affects expression	Homo sapiens	20044591
Nanotubes, Carbon	ELL2	increases expression	Mus musculus	25554681
Nanotubes, Carbon	ELL2	increases expression	Mus musculus	25554681
Nickel	ELL2	affects expression	Homo sapiens	14575637
Nickel	ELL2	affects expression|decreases reaction	Homo sapiens	14575637
Particulate Matter	ELL2	affects cotreatment|increases abundance|increases expression	Homo sapiens	29432896
Particulate Matter	ELL2	decreases expression	Mus musculus	32873817
Silicon Dioxide	ELL2	decreases expression	Homo sapiens	25895662
Sodium arsenite	ELL2	affects expression	Danio rerio	19590694
Sodium arsenite	ELL2	affects methylation	Homo sapiens	28589171
Sulforaphane	ELL2	increases expression	Homo sapiens	26833863
T-2 Toxin	ELL2	decreases expression	Gallus gallus	31299295
Tetrachlorodibenzodioxin	ELL2	affects expression	Homo sapiens	22298810
Tetrachlorodibenzodioxin	ELL2	increases expression	Homo sapiens	19684285
Tetrachlorodibenzodioxin	ELL2	affects expression	Mus musculus	21570461
Tetrachlorodibenzodioxin	ELL2	decreases expression	Mus musculus	19770486
Tetrachlorodibenzodioxin	ELL2	increases expression	Mus musculus	17035482
Tetrachlorodibenzodioxin	ELL2	decreases expression|increases response to substance	Mus musculus	25975270
Tetrachlorodibenzodioxin	ELL2	decreases expression	Rattus norvegicus	32109520
Tetrachlorodibenzodioxin	ELL2	increases expression	Rattus norvegicus	18796159
Tobacco Smoke Pollution	ELL2	increases expression	Homo sapiens	33660061
Tobacco Smoke Pollution	ELL2	decreases expression	Mus musculus	31705857
Vehicle Emissions	ELL2	increases methylation	Mus musculus	25560391

It is worth mentioning that Tobacco Smoke Pollution and Particulate Matter (PM), as highly accessible environmental toxins, are believed to concurrently upregulate the expression levels of DAPP1 and ELL2 in human species. Therefore, avoiding exposure to these toxins may improve UC patients’ therapeutic responsiveness.

In addition, we examined the relationship between certain drugs and DAPP1 and ELL2 using the CTD database (Table 3). In humans, Zoledronic Acid and Tretinoin were found to enhance the expression levels of DAPP1 and ELL2 genes, whereas Antirheumatic Agents were observed to decrease their gene expression. Therefore, during anti-UC therapy, co-administration of Zoledronic Acid and Tretinoin may potentially reduce the therapeutic responsiveness of UC patients, whereas the administration of Antirheumatic Agents may potentially enhance their therapeutic responsiveness. There is still a need for further research to understand the underlying mechanisms, optimize drug choice and dosage, and ultimately improve the management of UC.

**Table 3:** The interaction between drug exposure and DAPP1 and ELL2.

**Chemical name**	**Gene symbol**	**Interaction actions**	**Organism**	**Reference PubMedID**
Abrine	DAPP1	increases expression	Homo sapiens	31054353
Acetaminophen	DAPP1	decreases expression	Rattus norvegicus	33387578
Antirheumatic Agents	DAPP1	decreases expression	Homo sapiens	24449571
Choline	DAPP1	affects cotreatment|increases expression	Mus musculus	20938992
Dronabinol	DAPP1	decreases expression	Homo sapiens	29691375
Estradiol	DAPP1	increases expression	Homo sapiens	31614463
Ethinyl Estradiol	DAPP1	decreases expression	Xenopus tropicalis	23129252
Folic Acid	DAPP1	increases expression	Mus musculus	25629700
Folic Acid	DAPP1	affects cotreatment|increases expression	Mus musculus	20938992
Gentamicins	DAPP1	increases expression	Rattus norvegicus	33387578
Methionine	DAPP1	affects cotreatment|increases expression	Mus musculus	20938992
Pirinixic acid	DAPP1	affects binding|decreases expression|increases activity	Mus musculus	19710929
Tretinoin	DAPP1	increases expression	Homo sapiens	33167477
Zoledronic Acid	DAPP1	increases expression	Homo sapiens	20977926|24714768
Abrine	ELL2	increases expression	Homo sapiens	31054353
Acetaminophen	ELL2	decreases expression	Homo sapiens	26497421
Acetaminophen	ELL2	increases expression	Homo sapiens	21420995
Acetaminophen	ELL2	affects expression	Mus musculus	15606129|17562736
Acetaminophen	ELL2	increases expression	Rattus norvegicus	32479839|33387578
Antirheumatic Agents	ELL2	decreases expression	Homo sapiens	24449571
Choline	ELL2	affects cotreatment|decreases methylation	Mus musculus	20938992
Dronabinol	ELL2	increases expression	Rattus norvegicus	30283037
Estradiol	ELL2	decreases expression|decreases reaction	Homo sapiens	24758408
Estradiol	ELL2	decreases expression	Homo sapiens	24758408
Estradiol	ELL2	increases expression	Homo sapiens	31614463
Ethinyl Estradiol	ELL2	affects expression	Homo sapiens	20170705|26865667
Folic Acid	ELL2	affects cotreatment|decreases methylation	Mus musculus	20938992
Gentamicins	ELL2	decreases expression	Rattus norvegicus	22061828
Gentamicins	ELL2	increases expression	Rattus norvegicus	33387578
Methionine	ELL2	affects cotreatment|decreases methylation	Mus musculus	20938992
Pirinixic acid	ELL2	increases expression	Mus musculus	18445702
Tretinoin	ELL2	increases expression	Homo sapiens	21934132|33167477
Zoledronic Acid	ELL2	increases expression	Homo sapiens	20977926

### Validation of the causal relationship between DAPP1/ELL2 and UC via MR analysis

**Figure 9:**
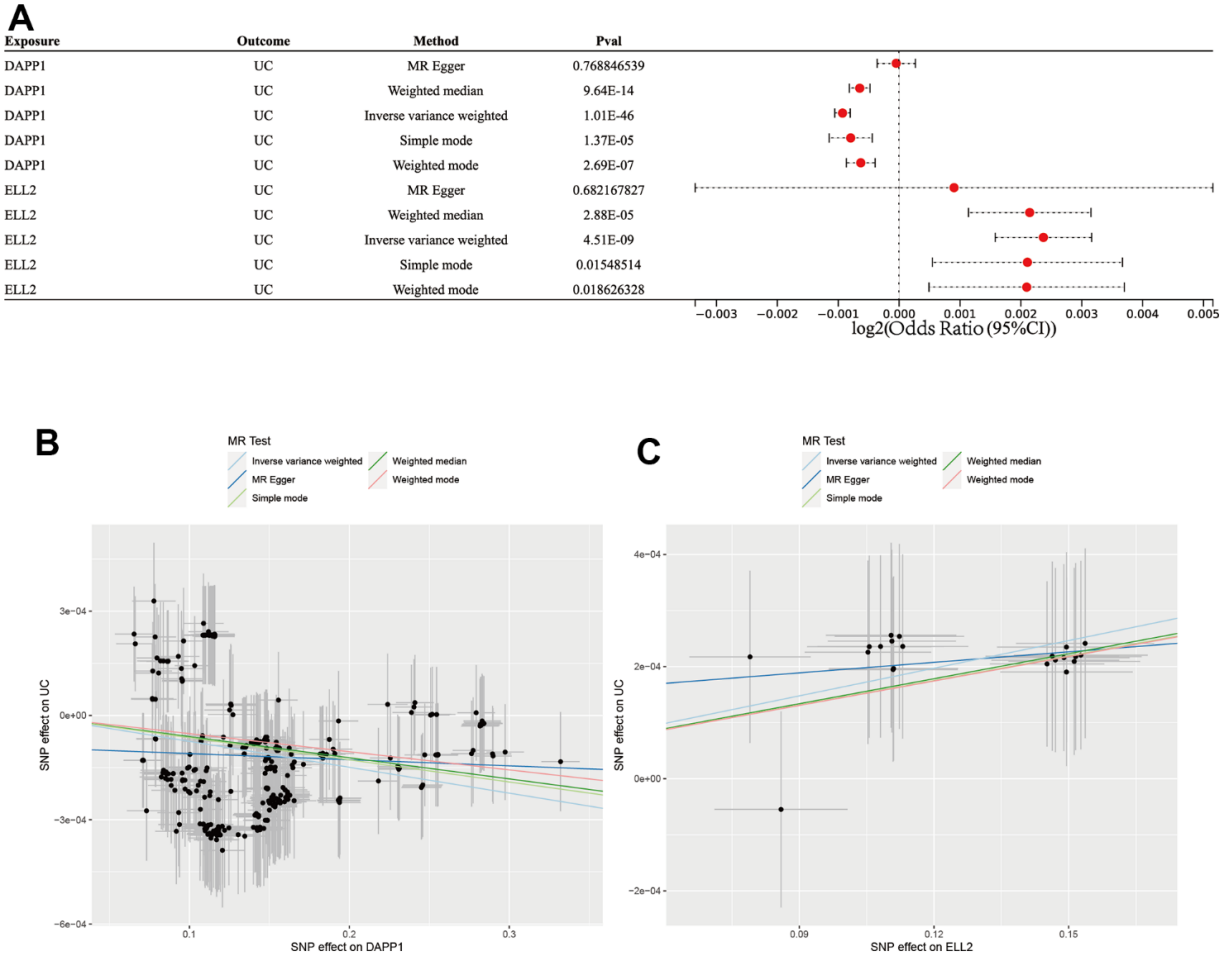
(**A**) Forest plot showing results from the Mendelian randomization analysis. (**B**) The scatter plot of five MR methods between DAPP2 and UC. (**C**) The scatter plot of five MR methods between ELL2 and UC.

Application of MR analysis integrated with GWAS and eQTL data facilitated the investigation of the association between UC and eQTL of DAPP1 and ELL2 (Figure 9A). Results obtained from the IVW, WM, MR-Egger methods, simple mode, and weighted model analyses all support that DAPP1 is a protective element against UC occurrence (IVW: p<0.001, WM: p<0.001, simple mode: p<0.001, and weighted model: p<0.001; Figure 9B). In addition, the analyses revealed a causal relationship between ELL2 and UC occurrence (IVW: p<0.001, WM: p<0.001, simple mode: p=0.02, and weighted model: p=0.02; Figure 9C).

### Using an independent validation set and RT-qPCR analysis for the validation of DAPP1 and ELL2 expression levels

To validate the link between DAPP1, ELL2, and cigarette pollution, an independent validation set, GSE72163, was employed. GSE72163 investigates the changes in the gene expression of intestinal epithelial cell line (DLD-1) and T-lymphocyte cell line (Jurkat) following exposure to cigarette smoke extract. cigarette smoke extract did not cause a significant change in the expression of ELL2 gene in Jurkat cells. However, both DLD-1 cells and Jurkat cells exposed to cigarette smoke extract exhibited a significant downregulation in DAPP1 expression levels (Supplementary Figure 6). Previous studies have suggested that smoking may prevent the occurrence and reduce the severity of ulcerative colitis [51]. However, our study found that DAPP1 was upregulated in UC patients with intestinal inflammation. Therefore, the results obtained from the independent validation set GSE72163 are consistent with our findings.

## DISCUSSION

In this study, we retrieved and incorporated 4 microarray queues of patients who received treatment with 5-ASA, corticosteroid, golimumab, or infliximab, and demonstrated treatment responsiveness over an explicit four-week follow-up period. We subsequently conducted a Pan-therapy Analysis of UC. Through this bioinformatic and machine learning-based Pan-therapy Analysis, we have obtained two key genes, namely DAPP1 and ELL2, that hold the potential to influence the response to all the aforementioned four pharmacotherapies of UC. The specific research process can be found in Supplementary Figure 7.

It is worth noting that this study represents the first time that the study of DAPP1 and ELL2 has been linked to UC, indicating potential avenues for further research. DAPP1, also known as B cell adaptor molecule of 32 kDa (Bam32), has been proposed to regulate lymphocyte proliferation and recruitment during inflammation [52–54]. During the process of neutrophil recruitment, DAPP1-dependent, ERK1/2-involving ROS generation in neutrophils is deemed crucial in inducing WKYMVm-induced microvascular hyperpermeability [52]. In UC, areas infiltrated by neutrophils witness mucosal damage [55, 56]. Neutrophil extracellular traps (NETs), released by neutrophils, also play a vital role in sustaining mucosal inflammation in UC [57–59]. These findings highlight the potential of DAPP1 to modulate inflammation in UC, consistent with our own research indicating elevated expression of DAPP1 in the inflamed gut of UC patients. The research reveals that Bam32-/- mice exhibit impaired innate B cells, leading to increased susceptibility to infections. This is mainly due to a significant decrease in serum levels of specific IgG, especially IgG1 and IgG2a classes, in Bam32-/- mice post-infection, while IgM antibody levels remain unaffected [60]. Previous studies have suggested that the sustained efficacy of biologic therapies in autoimmune diseases like pemphigus is linked to a lasting depletion of IgG-switched memory autoreactive B cells. This, in turn, leads to the disappearance of antibody-secreting cells [61]. In addition, B cells encode focused antibody repertoires that include antibodies that stimulate macrophage TNF-α production [62–64]. Incidentally, TNF-α is one of the therapeutic targets for treating UC, for instance, through golimumab [65, 66]. Therefore, the regulation of the DAPP1-B cell-TNF-α axis may be one potential mechanism for the therapeutic response in UC. In the context of our study, the positive correlation between DAPP1, B cells, and iTreg cells lends some plausibility to this hypothesis. It has been reported that regulatory T cells exhibit minimal levels of effector cytokine expression, thereby suppressing immune responses and inflammatory processes [67–69]. Elevated levels of Treg cells have been noted in ulcerative colitis and colon cancer. Following T-cell receptor engagement, colitic Treg cells produce high amounts of IL-8, further demonstrating their importance as IL-8 producers within the gut [67]. A retrospective analysis involving 271 UC patients and healthy controls concluded that IL-8 could serve as an excellent biomarker for predicting the severity of UC [67]. By modulating inflammatory cytokines like IL-8, 5ASA can relieve inflammation of the UC intestinal mucosa. Therefore, higher levels of ELL2 may lead to increased infiltration of Treg cells and subsequent production of IL-8. This could partly explain how ELL2 may affect the efficacy of UC treatment.

Hence, targeting DAPP1 and ELL2 has the potential to be a promising approach to improving response rates to current UC treatment regimens. It is worth noting that DAPP1 and ELL2 are excellent biomarkers for UC in its inflammatory stages, making them potential targets for slowing down the progression of UC-associated inflammation. In this regard, we used the XSum algorithm to identify potential small molecule drugs from the CMAP database that could alleviate the inflammatory course of UC. Among these molecules, Clofibrate was subjected to molecular docking with DAPP1 and ELL2, and the relatively impressive docking scores suggest that it can bind well with the DAPP1 and ELL2 proteins. Nevertheless, we must approach these findings with caution, as the identification of potential drugs was achieved through computational methods. In-depth mechanistic studies are necessary to validate these results rigorously. In summary, we have identified two therapeutic targets that affect treatment response in UC patients, and this effect is not limited to only one single treatment modality. These results may pave the way for subsequent solutions to combat drug resistance or lack of response to medication in UC patients.

In this study, we further employed MR analysis to explore the causal relationship between essential genes and UC incidence. Mendelian randomization, a technique that utilizes genetic data to investigate causal relationships between variables, was utilized. Our findings indicate that despite the up-regulation of DAPP1 and ELL2 in the inflammatory sites of UC, MR analysis revealed that DAPP1 up-regulation serves as a protective factor against UC, whereas ELL2 up-regulation is linked to the onset of UC. As previously reported, DAPP1 plays an essential role in the adhesion, migration, and recruitment of neutrophils, and excessive neutrophil infiltration is one of the reasons for the elevated levels of UC inflammation [52, 57, 70]. However, it is worth noting that the recruitment of neutrophils is beneficial for eliminating invading intestinal pathogens, which is crucial for maintaining a balanced intestinal microbiota [71–73]. Imbalance of intestinal microbiota plays a role in UC pathogenesis [3, 74–76]. Therefore, DAPP1 serves as a preventive factor in the initial stage of UC disease, while excessive recruitment of neutrophils mediated by DAPP1 aggravates UC inflammation during the course of the disease. Furthermore, we noted that ELL2 plays a consistent role in both the incidence and inflammation of UC, highlighting the promising potential of targeting ELL2 for drug development and validation in the treatment and management of UC. However, the present study did not further explore whether the ability of DAPP1 and ELL2 to predict treatment response in UC is due to their regulation of inflammation in the intestinal tract of UC patients, which is also one of the limitations of the study.

It is worth mentioning that Tobacco Smoke Pollution and Particulate Matter, as highly accessible environmental toxins, are thought to simultaneously upregulate the expression levels of DAPP1 and ELL2 in humans. In addition to increasing the likelihood of developing and exacerbating CD, smoking reduces the severity of UC and protects against its onset [51]. It remains unclear whether smoking elicits any influence on the therapeutics’ effectiveness in treating IBD. However, there are some limited clinical data that suggest Tobacco Smoke may exert deleterious effects on the therapeutic efficacy of certain drugs for IBD. Smokers who receive infliximab for IBD manifest markedly lower median trough levels as compared to nonsmokers [77]. Moreover, smokers were observed to exhibit significantly elevated levels of anti-infliximab antibodies as compared to nonsmokers, thereby suggesting a compromised response towards infliximab in IBD from a pharmacokinetic perspective [77]. Simultaneously, previous research implied that smoking is also an independent risk factor for augmenting the likelihood of terminating thiopurine-based therapy owing to unfavorable impacts [78]. The ingestion of PM emanating from cigarette smoke causes gastrointestinal dysfunction, leading to disturbance of mucus secretion, mucosal microcirculation, and mucous repair processes in the intestinal tract [78]. Not only this, roughly 85% of lung cancers are attributed to cigarette smoking, whereas the remaining fraction is accredited to passive smoking, which non-smokers are exposed to [79]. In addition, the relationship between cigarette smoke and immune cells has been widely investigated. Under inflammatory conditions, cigarette smoke alters the peptide repertoire of antigens on MHC class I molecules. Importantly, activation of IAV-specific CD8+ T cells mediated by MHC class I is inhibited by cigarette smoke [80]. Cigarette extract leads to activation of ROCK2 and reduced phosphorylation of IRF4 in T cells. This effect is associated with an increased production of IL-22 [81]. The above evidence suggests a close relationship between cigarette smoke and T cells, whereas T cells are closely linked to the occurrence and progression of UC. Therefore, our investigation suggests that UC patients should avoid Tobacco Smoke Pollution during their treatment regimen. Another interesting finding of our study is that PM is also responsible for affecting the treatment response of UC, a scenario that has not been previously explored in the literature. The ingestion of PM and cigarette smoke pollution can cause gastrointestinal dysfunction, leading to disturbance of mucus secretion, mucosal microcirculation, and mucous repair processes in the intestinal tract [82, 83]. In addition, it is worth mentioning that PM in the ambience was recently categorized as a Group I carcinogen by the International Agency for Research on Cancer (IARC) [84]. Hence, a salubrious air quality may be advantageous for UC patients during the course of their treatment.

This investigation has provided innovative concepts and resources for individualized clinical regimens for those afflicted with UC, although certain limitations of the present inquiry must be acknowledged. The study only included bioinformatics analysis, with limited experimental validation to provide a robust foundation. Limitations in the availability of microarray datasets meeting the inclusion criteria resulted in the restriction of only four therapeutic modalities and corresponding treatment response states being analyzed in the Pan-therapy Analysis of UC as presented in this study. Consequently, future research endeavors must remain focused on obtaining more diverse treatment modalities to enable further in-depth exploration. Furthermore, one of the study’s drawbacks is its retrospective nature, rather than a prospective trial. Hence, further follow-up investigations utilizing mechanistic exploration and prospective clinical trials are crucial to validate our findings.

## CONCLUSIONS

We utilized microarray technology to conduct a Pan-therapy Analysis and identify two key gene signatures (DAPP1 and ELL2) as biomarkers of unresponsiveness to multiple therapies and inflammatory progression in IBD. Our research also identified Clofibrate as a potential small molecule drug for UC treatment and comprehensively reviewed environmental toxins and drug exposures that may affect UC responsiveness. This contribution enables personalized clinical management and treatment regimens for UC.

## Supplementary Material

Supplementary Figures

Supplementary Table 1

Supplementary Table 2

Supplementary Table 3

Supplementary Table 4

Supplementary Table 5

Supplementary Table 6

Supplementary Table 7
