# Supplementary material for: Comprehensive analysis reveals key genes and environmental toxin exposures underlying treatment response in ulcerative colitis based on in-silico analysis and Mendelian randomization
Source: Aging (Albany NY). 2023 Dec 4;15(23):14141–71. doi: 10.18632/aging.205294 (PMC10756092; doi:10.18632/aging.205294)
Supplement: Supplementary Figures [file aging-15-205294-s001.pdf]

SUPPLEMENTARY FIGURES

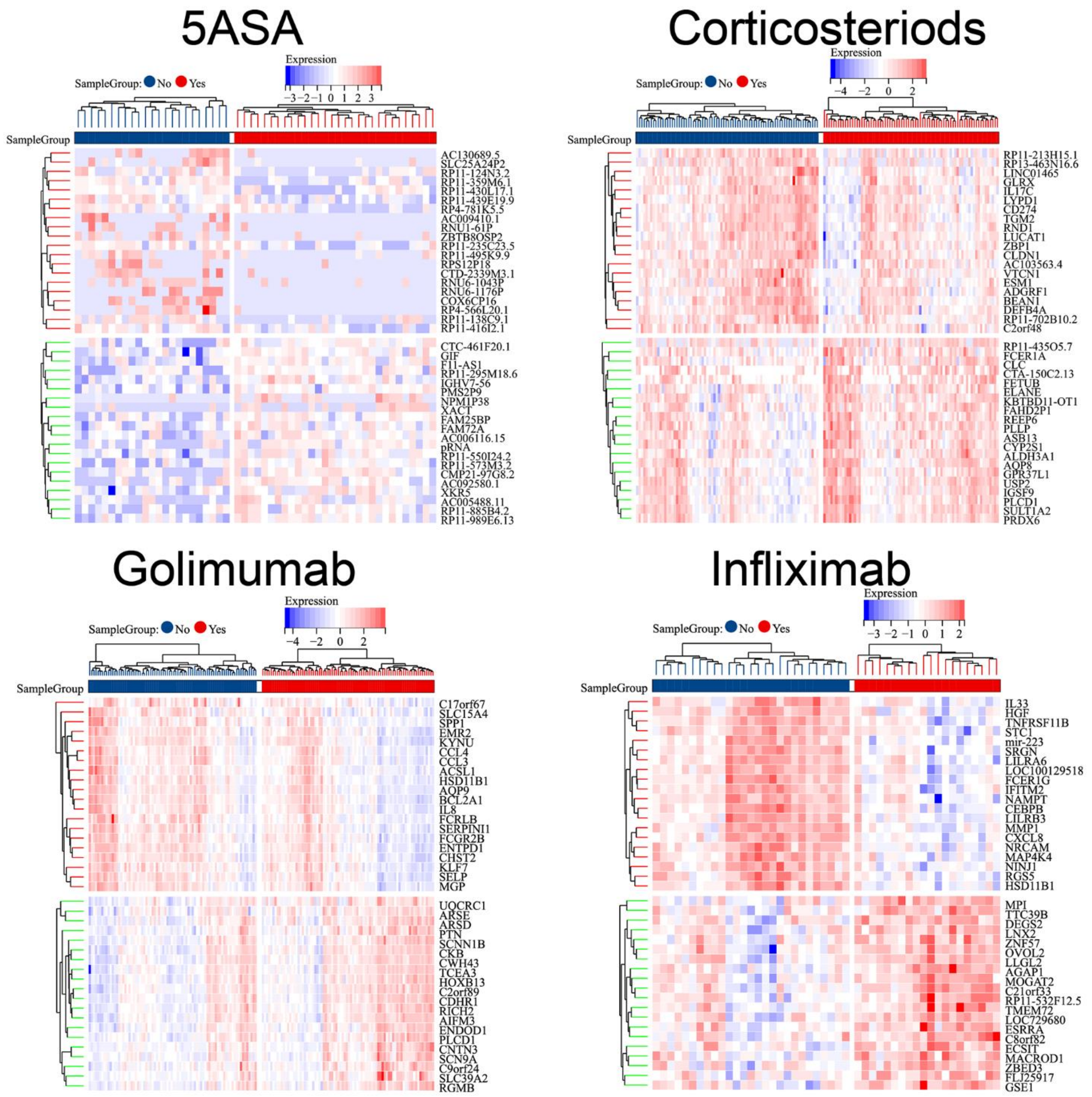

Supplementary Figure 1. Heatmap illustrating the top 20 genes that were differentially up-regulated and down-regulated in the UC patients who do not respond to 5-ASA (top left), corticosteroids (top right), golimumab (bottom left), or infliximab (bottom right) treatment.

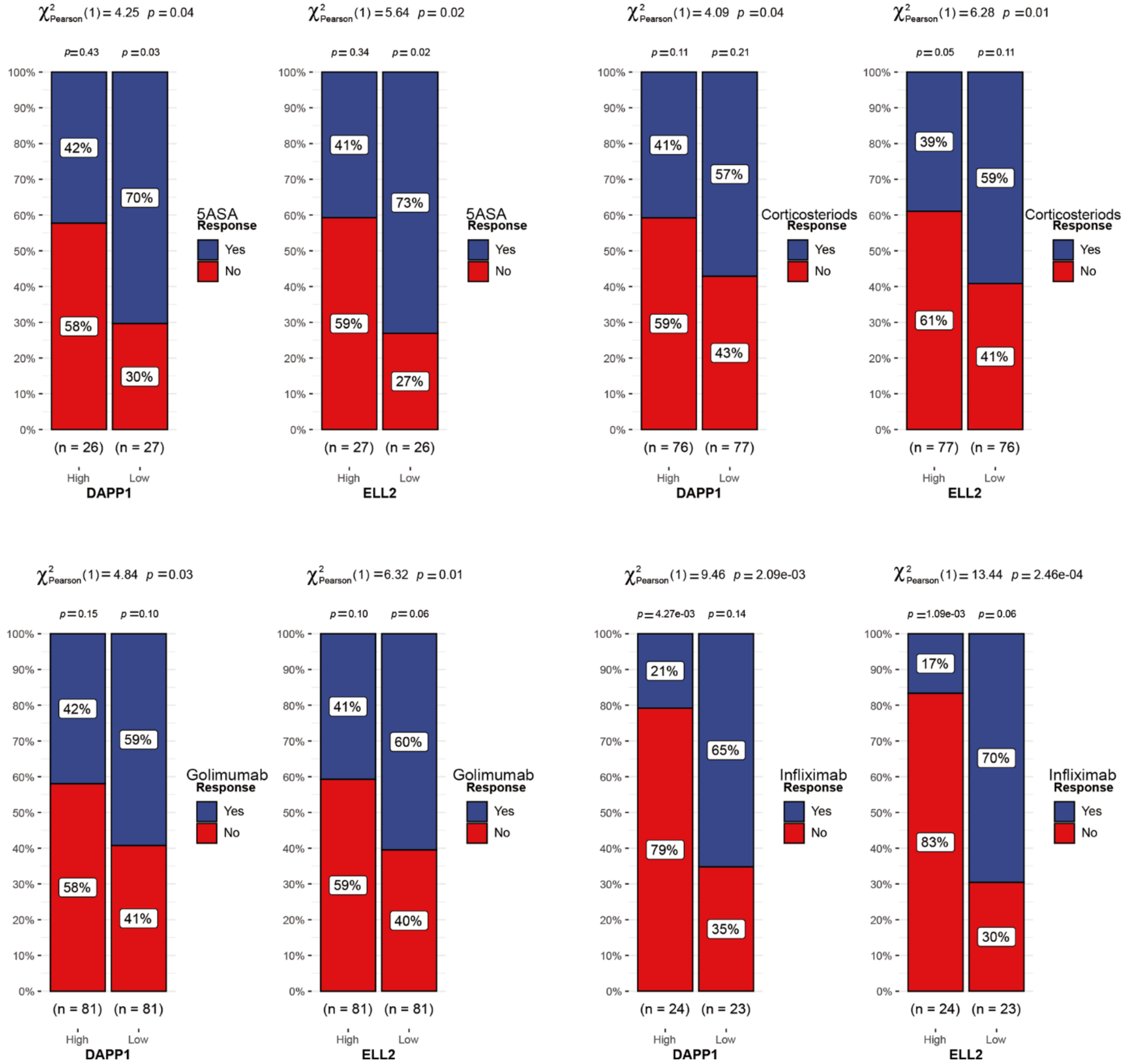

Supplementary Figure 2. The proportion of patients who respond or do not respond to 5-ASA, corticosteroids, golimumab, or infliximab treatment varies with high and low levels of DAPP1 and ELL2 gene expression.

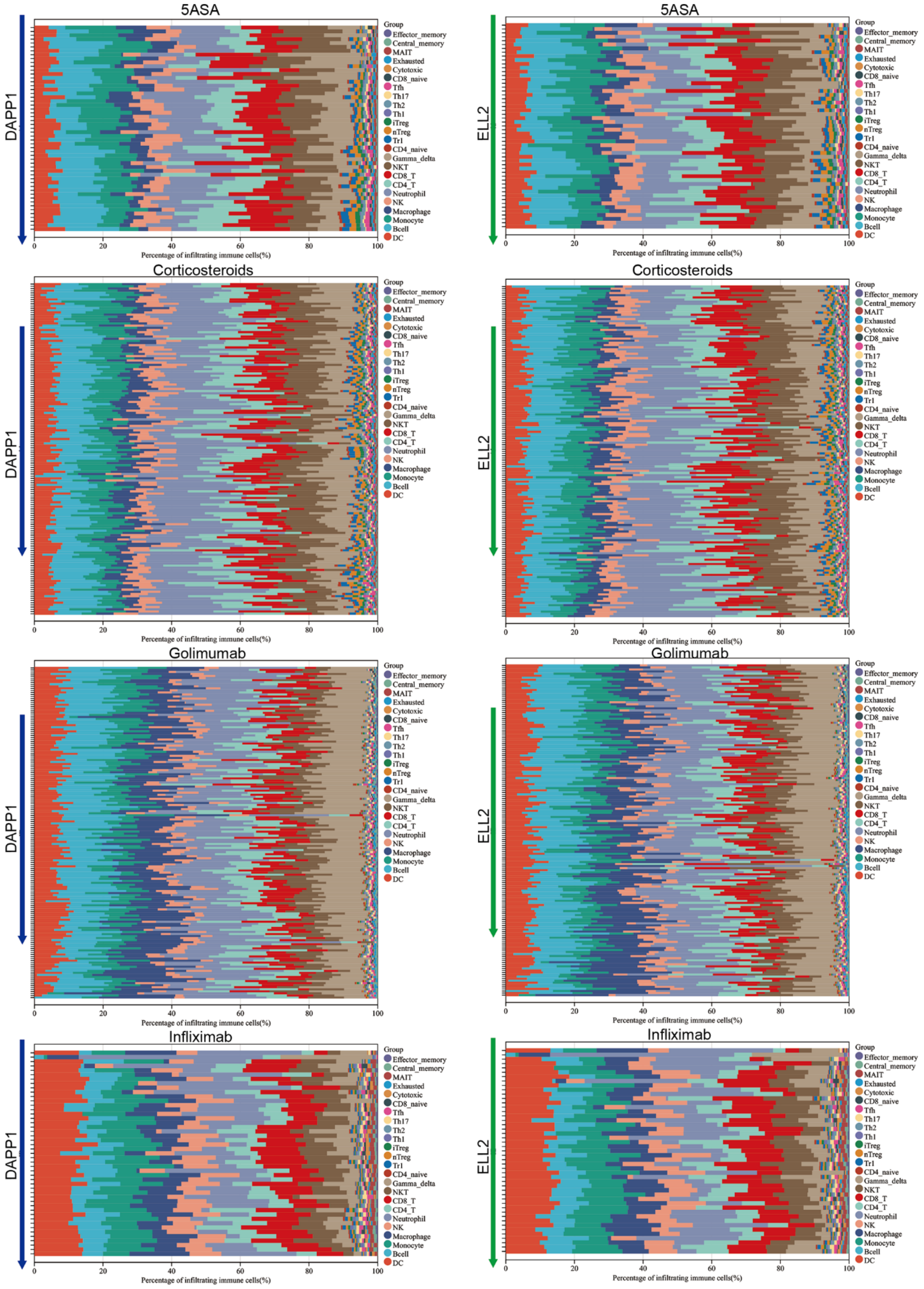

Supplementary Figure 3. The immunological cell infiltration landscape in samples from treatment cohorts with 5-ASA, corticosteroids, golimumab, or infliximab changes as the gene expression levels of DAPP1 and ELL2 are upregulated.

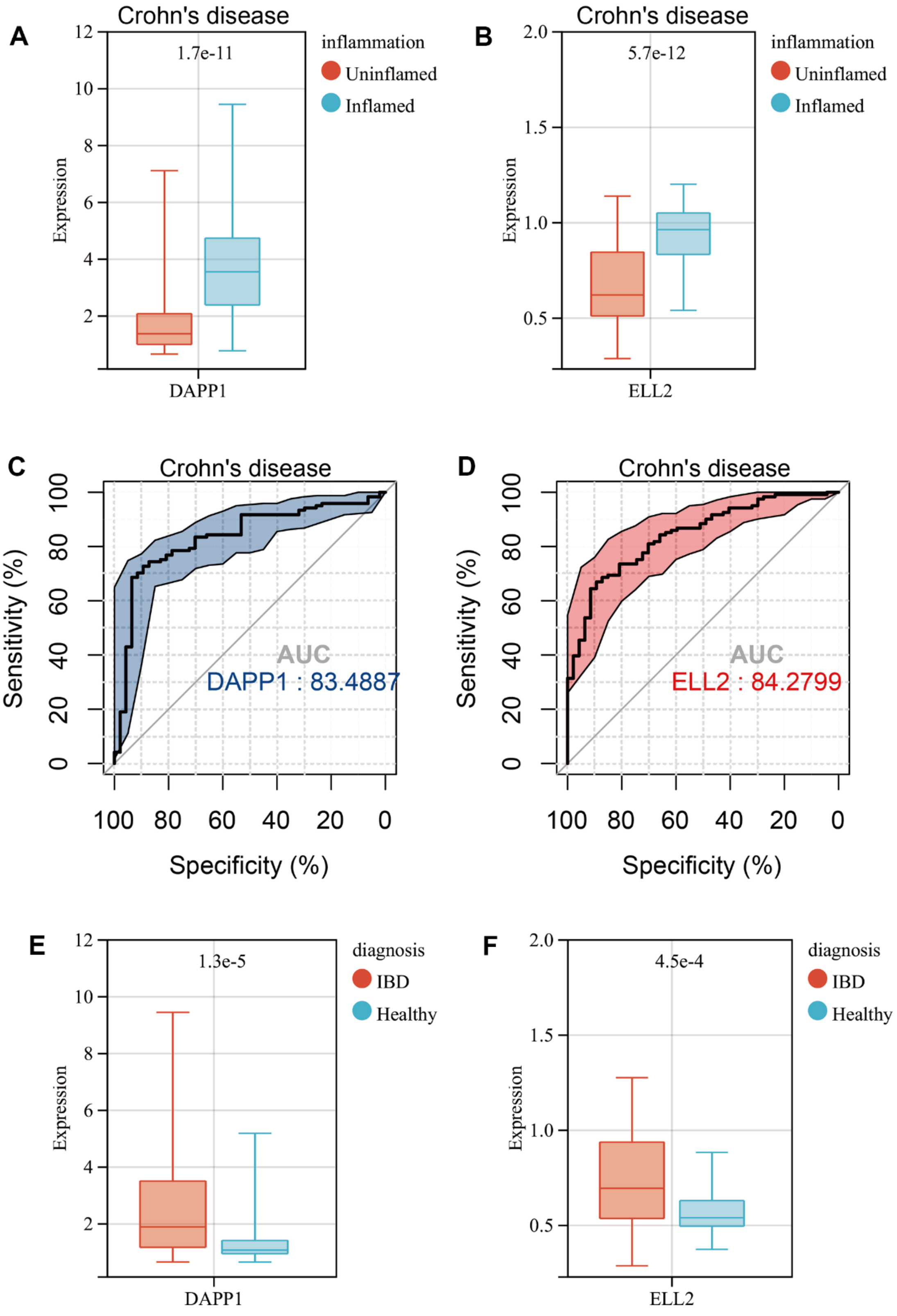

Supplementary Figure 4. Boxplots of the gene expression levels of DAPP1 (**A**) and ELL2 (**B**) in inflamed and uninflamed Crohn's disease samples in GSE179285 cohort. AUCs of DAPP1 (**C**) and ELL2 (**D**) in ROC analysis predicting Inflamed Crohn's disease samples. Boxplots of the gene expression levels of DAPP1 (**E**) and ELL2 (**F**) in IBD samples and Healthy controls.

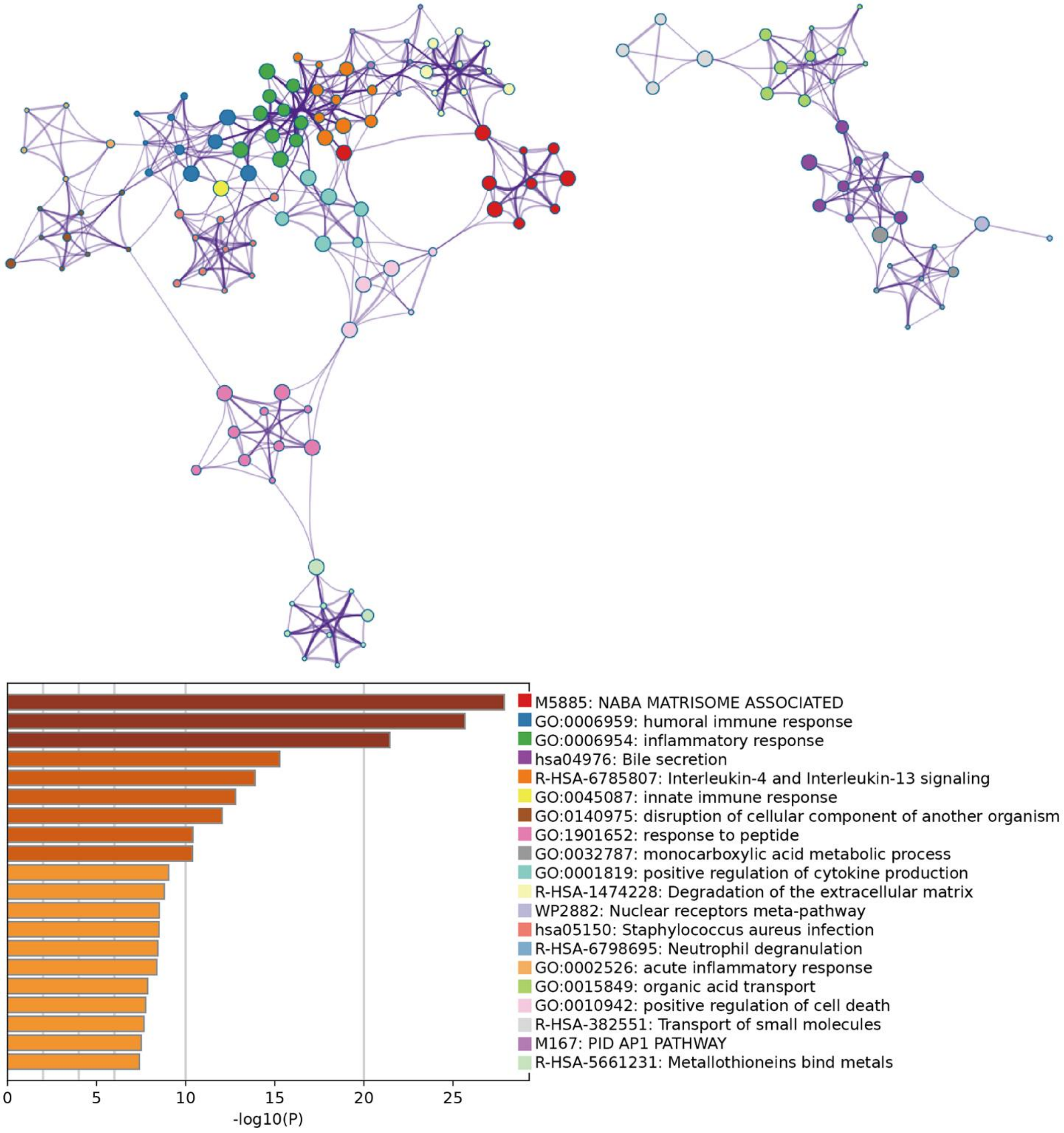

Supplementary Figure 5. Enrichment analysis results of the top 150 upregulated and top 150 downregulated genes in Inflamed UC based on the Metascape database.

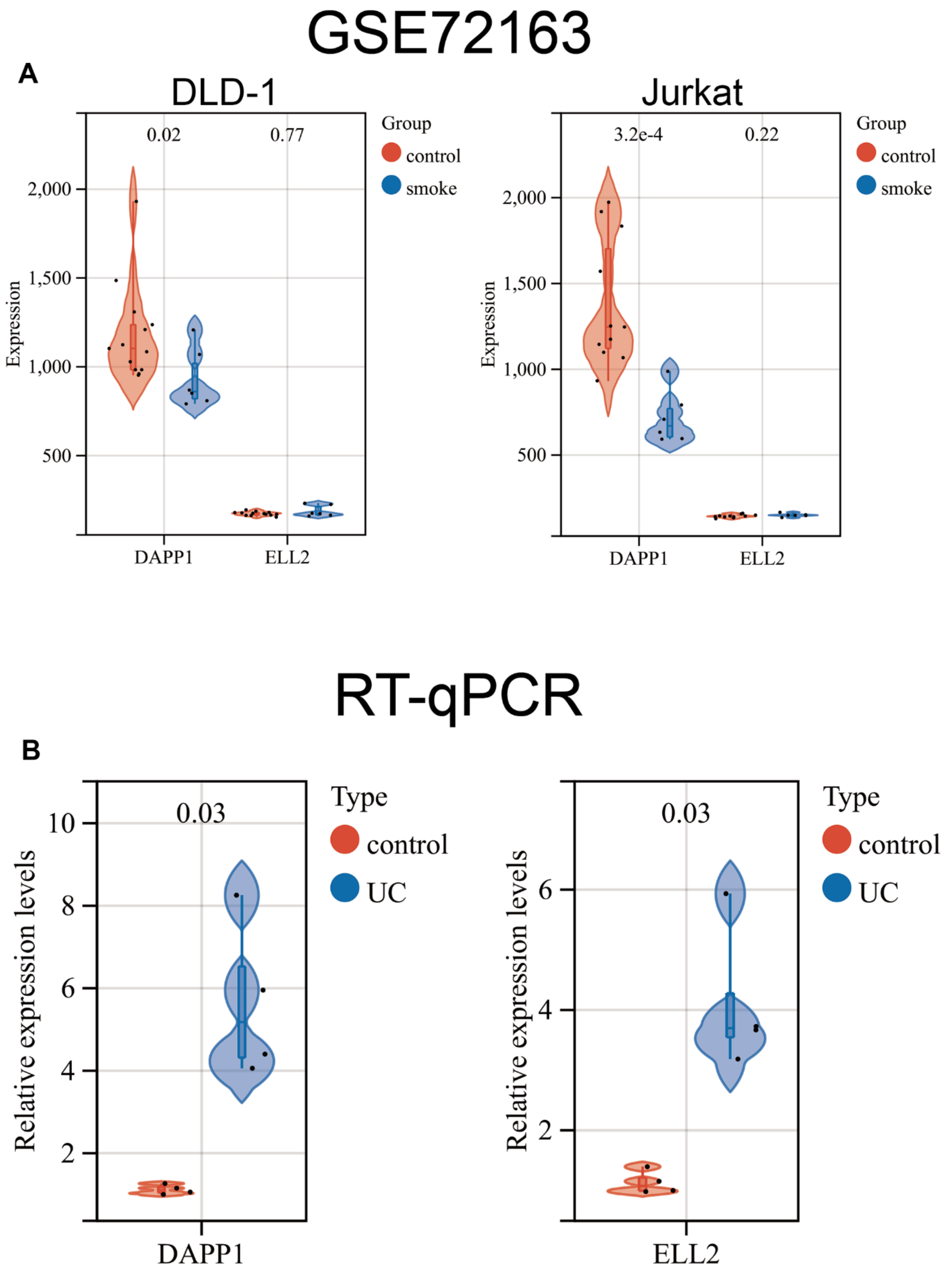

Supplementary Figure 6. (**A**) Changes in the gene expression levels of DAPP1 and ELL2 in DLD-1 cells and Jurkat cells exposed to cigarette smoke extract. (**B**) The RT-qPCR analysis validated the differential expression of DAPP1 and ELL2 in the intestinal mucosa of UC patients and healthy controls.

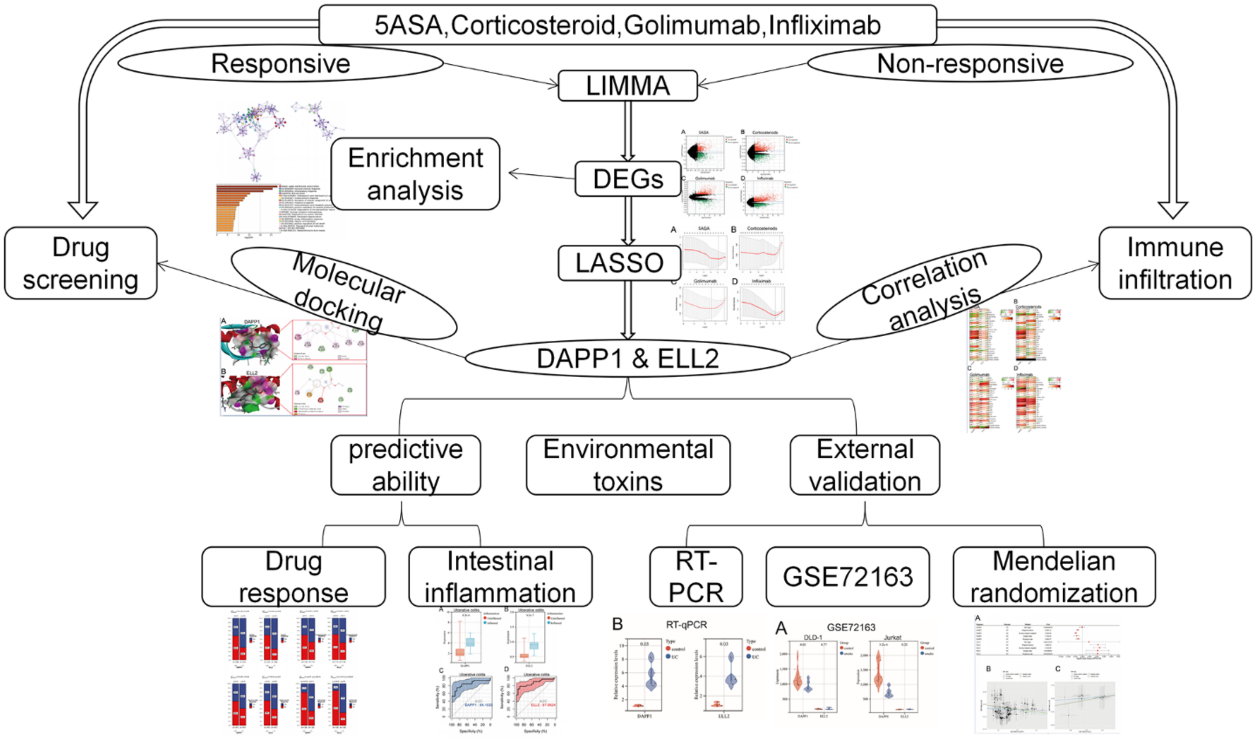

Supplementary Figure 7. The flowchart of this study.
