# Supplementary material for: Comprehensive analysis reveals key genes and environmental toxin exposures underlying treatment response in ulcerative colitis based on in-silico analysis and Mendelian randomization
Source: Aging (Albany NY). 2023 Dec 4;15(23):14141–71. doi: 10.18632/aging.205294 (PMC10756092; doi:10.18632/aging.205294)
Supplement: Supplementary Table 2 [file aging-15-205294-s003.pdf]

Supplementary Table 2. Lasso analysis results in 5-ASA, corticosteroid, golimumab, and infliximab treatment cohort.

Treatment	Lasso genes
5ASA	DAPP1<br>ELL2<br>IGSF6<br>SAMD9L
Corticosteriods	DAPP1<br>ELL2
Golimumab	DAPP1<br>ELL2<br>LYN<br>SAMD9L
Infliximab	DAPP1<br>ELL2<br>IGSF6<br>LYN
